# Perceptions of females about trauma-informed services for survivors of sexual violence in south western Uganda- a qualitative study

**DOI:** 10.1186/s12889-021-12227-0

**Published:** 2021-11-23

**Authors:** Earnest Amwiine, Bonita Ainembabazi, Isaiah Obwona, Richard Opoka, Mary Akatuhumuriza, Vallence Niyonzima, Vincent Mubangizi

**Affiliations:** Faculty of Medicine, Mbarara University of Science and Technology, P.O Box 1410, Mbarara, Uganda

**Keywords:** Sexual violence, Trauma-informed services, Perception, Attitude, Knowledge, Uganda

## Abstract

**Background:**

Sexual violence is a public health concern globally and locally, and trauma-informed services are put in place to mitigate its consequences. A few studies have evaluated the quality and uptake of these trauma-informed services for sexual violence. This study aimed at; i) assessing the knowledge of participants about trauma-informed services, ii) exploring the attitudes of females about trauma-informed services, and iii) assessing different factors associated with the utilization of trauma-informed services.

**Methods:**

This study employed a descriptive cross-sectional qualitative design. The participants were females at Kyangyenyi health center III and Kigarama health center III in Sheema district, southwestern Uganda. We used a purposive sampling procedure for all participants and then a consecutive sampling of females. Data about; knowledge of trauma-informed services for sexual violence, attitudes towards trauma-informed services, and factors influencing the utilization of trauma-informed services were collected using an in-depth interview guide. Data were analyzed using thematic content analysis.

**Results:**

We interviewed 32 participants. There was a high prevalence of sexual violence, and it was a big concern in the community. Many of the respondents were not sensitized about trauma-informed services. Most of them knew only about HIV treatment. Our study shows that a good section of females did not seek the services after being sexually violated due to the fear of the perpetrator, bribing of the family of the affected and authorities, or even fear of family breakup and stigmatization. There were a lot of bribery, poor road networks, and inadequate health care services. These hindered survivors of sexual violence from utilizing trauma-informed services.

**Conclusions:**

There was a knowledge gap about trauma-informed services for survivors of sexual violence. There was sexual violence in the community. Sensitization needs to be done among the populations by respective authorities to iron out issues of ignorance about the services and health workers evaluated for competence in offering the trauma-informed services.

## Background

Sexual violence is a major public health problem globally. The prevalence of partner and non-partner sexual violence in women is as high as 59 and 6% respectively, in some regions [1–3]. Twenty-two percent of females aged 15–49 years in Uganda have experienced sexual violence [4]. Sexual violence was common among human immunodeficiency virus (HIV) positive women at 28.3% [5].

Trauma-informed care or services is a strength-based framework. It increases providers’ awareness and understanding of the impact of trauma, thus guiding and monitoring their interventions, actions, and behaviors in working with clients and creating opportunities for survivors to rebuild a sense of control and empowerment, and minimizes any risks of re-traumatizing clients [6, 7]. Trauma-informed services ensure safety, empowerment, choice, and meeting the unique needs of trauma survivors, so they are more active participants in their care and recovery [8, 9]. Studies have shown that women with a history of sexual violence desire care and treatment of their unique needs from trauma-informed health care providers [10, 11]. The trauma-informed care framework has been implemented successfully for survivors of sexual violence in primary and mental healthcare settings [11–13], women clinics [14], and perinatal care settings [15]. The use of trauma-informed services for survivors of sexual violence has been documented in Kenya [16].

The effects of sexual violence range from psychological, physical, social, and reproductive health problems [17–20]. All these negatively impact the health of individuals and so needs to be addressed to improve livelihoods. Uganda has formulated policies and laws against sexual violence and has been evaluated over time to see their effectiveness [20, 21]. A package of trauma-informed services should be provided to survivors of sexual violence so as not to re-traumatize the survivors.

The components of trauma-informed services for survivors of sexual violence in Uganda include HIV testing, emergency antiretroviral therapy, emergency contraception, sexually transmitted infections testing, counseling services, tetanus toxoid, provision of prophylactic antibiotics, medico-legal examination, and provision of a medico-legal certificate, and referrals to other organizations for social support [22]. These components are adapted from the World Health Organization guidelines and should be available at all health facilities from health center three and above. The survivors of sexual violence go back to their residence, where they may not get a safe shelter. Some health service providers at these health facilities were trained on trauma-informed services to survivors of sexual violence [20]. These services are provided in a way that avoids exacerbating existing trauma, prevents creating additional trauma, empowers the client, promotes recovery, and respects rights.

Few survivors of sexual violence can access or even complete the necessary health care services [23]. According to Uganda’s demographic and health survey 2011, up to 42% of sexual violence victims did not seek medical assistance [24]. Also, most survivors of sexual violence did not go to health facilities first [24]. Even when survivors of sexual violence assessed health facilities, a big percentage did so late after 24 h after the act of sexual violence [25]. This delay exposes them to an increased likelihood of developing different health conditions.

There is a paucity of studies on trauma-informed services in Uganda. The study explored the knowledge, attitudes, and perceptions of females, and factors associated with the accessibility of trauma-informed services for survivors of sexual violence. Understanding these factors in a resource-constrained setting, like Uganda, is crucial in influencing policy recommendations and creating public awareness about improved trauma-informed service delivery.

## Methods

### Study design

This study was a descriptive cross-sectional design employing a qualitative approach conducted in April 2021.

### Study setting

The study was carried out at selected health facilities of Kyangyenyi health center III and Kigarama health center III in Kyangyenyi and Kigarama sub-county respectively in Sheema district. In Uganda, health center IIIs do not have general doctors but have clinical officers, nurses, and midwives. Sheema district is bordered by Buhweju district to the north, Mbarara district to the east, Ntungamo district to the south, Mitooma district to the southwest, and Bushenyi district to the west. The district headquarters are approximately 33 km west of Mbarara city along Mbarara Kasese road. The projected district population was 220,500 whom 114,400 were females, and 106,100 were males [4]. Subsistence agriculture, animal husbandry, and petty trading are the main economic activities in the area [26]. People have water and food insecurity [27]. Among the challenges faced in the area like other areas in Uganda is the limited number of health workers [28] and the fact that health providers are well known, or members of the community, and confidentiality and providers’ fear of retaliation by alleged perpetrators or their families poses a challenge [29].

### Study population and sampling

Our study population was females attending Kyangyenyi health center III and Kigarama health center III in the Sheema district. The study employed purposive random sampling to ensure that participants represented all age categories; adolescents aged 15–19 years, young adults aged 20–30, middle-aged women 31–49, and the elderly above 50 years. Females were categorized into different age groups because we wanted to get a wide range of views from all age groups since each category faces challenges regarding sexual violence. At each health facility, we recruited four participants from each age category. In total, there were 32 participants for in-depth interviews.

### Interviewers

Two social research assistants conducted the interviews. They had experience in qualitative data collection. They underwent training on how to use the research tools for 1 day. The interviewers had not established any relationships with the study participants before data collection.

### Data collection tool

We specifically developed interview guides to collect data for this study based on literature. The interview guides were pre-tested among eight participants, and the results were used to adjust and validate the tools. Interview guides were written in English, translated from English into the local language (Runyankore-Rukiga), and then back-translated to verify fidelity to the original wording. Data were collected through in-depth face-to-face interviews using open-ended questions, followed by probes with the study objectives. The interview guides collected data on the socio-demographic characteristics of the participants, understanding of sexual violence, awareness about sexual violence in the community, knowledge about trauma-informed services, and the perception of these services.

### Data collection procedure

We obtained administrative approval from the district health officer and in-charge of health facilities. Different females attending the facilities on days of data collection were approached, the study was explained to them and invited to participate. The health provider at the health facility introduced the interviewers to potential participants. The potential participants were informed that we wanted their perceptions about trauma-informed services for survivors of sexual violence in the community. We did not specifically target survivors of sexual violence through some of the participants had experienced sexual violence. All those we asked to participate, accepted. Participants were 18 and above years old. We obtained written consent from participants.

Research assistants interviewed participants face to face, using a semi-structured interview guide. The interviews were conducted in a quiet place at a health facility. The in-depth interviews were conducted in the local language (Runyankore-Rukiga). The interviews were audio-recorded and backed by field notes with the participant’s consent. Each interview, lasting on average 30 min, was conducted until no new information was being generated.

### Quality control

We piloted our data collection tools at a separate health center for clarity and usability. The research assistants were trained on study objectives, informed consent, sexual violence, trauma-informed services, and data collection procedures. There were feedback meetings (both physical and virtual) between research assistants and VM at the end of each day of data collection, to check for completeness of the data. RO and VM attended some of the interviews to ensure that interviews were conducted according to the study objectives. Transcribing was done in the local language.

### Data management and analysis

Interviews were audio-recorded on a digital voice recorder notes were taken during the interviews. At the end of the data collection, recorded interviews were transcribed verbatim into Microsoft word documents. The transcripts in the local language were translated into English by a different person who was not involved in interviewing the participants to ensure quality. The transcripts were cleaned and anonymized. Processed data were entered into Atlas. ti version 7.5.7 (qualitative analysis software for Windows) for coding and analysis. After internalizing data a coding frame was developed, and agreed upon by three researchers (EA, VN, and VM). Data were coded. Data were analyzed using thematic content analysis.

### Ethical considerations

Mbarara University of Science and Technology Research Ethics Committee gave ethical approval (30/11–20). The Uganda National Council for Science and Technology granted the study clearance in line with national guidelines. We also obtained administrative approval from the district health officer and health center administrators. All participants were provided with explanations about the study and requested to provide written informed consent. In the case of illiterate participants, they used a thumbprint, and an independent witness signed to confirm that they consented freely. Participants provided written consent. Participation was voluntary, and a participant was free to withdraw from the study at any time without any penalties. Privacy and confidentiality were ensured by conducting the interviews in private and allocating non-identifiable field codes to each participant.

## Results

Three themes emerged during the analysis: a) knowledge of females about sexual violence, b) knowledge about trauma-informed services, and c) attitudes towards trauma-informed services. The study employed purposive random sampling to ensure that respondents represented all age categories. A total of 32 in-depth interviews using an interview guide were conducted. The mean age of participants was 32.9 years. Twenty-five of these were married, three were widowed, two were divorced, and two were single. Seventeen of the participants did not complete primary level of education, seven completed primary level, three completed senior four, and only four completed senior six of whom two were diploma holders, and one was a bachelor’s degree holder.

### Knowledge of participants about sexual violence

This section of the manuscript describes the understanding of the participants about sexual violence and its different forms, awareness about the situation of sexual violence in the communities, and the effects of sexual violence. Understanding sexual violence demystifies the trauma-informed services for survivors of sexual violence in the community. Also, understanding what sexual violence and its various forms have a bearing on whether survivors of sexual violence will seek the services.

#### Understanding of sexual violence by participants

Participants viewed sexual violence as an act of men forcing them into sexual acts. Other participants understood sexual violence as children raped by their fathers, relatives, and sometimes strangers. A husband forcing his wife into having sex was sexual violence. Participants also understood sexual violence with examples like someone touching their buttocks and breasts when someone has not consented.

*“Sexual violence means that me as a woman, I am not interested in being engaged in sexual activities, and yet you find a man is seriously using force to make me get involved when I am not interested.” P33: IDI_KYA_12*

*“Sexual violence is engaging someone in sexual acts without their consent, or asking someone to have sex with you, and when she refuses, you force her and do whatever you wanted.” P12: IDI_KIG_30*

Participants mentioned diverse forms of sexual violence in the community. These included intimate partner sexual violence, fathers/ guardians raping and defiling daughters, and women raped. Another form of sexual violence was men touching a lady’s private parts such as breasts without her consent which was more pronounced among superiors to their subordinates.

*“Husbands forcefully want to have sex with us, and when we refuse, they fight with us and even end up beating us and hurting us very badly. These men even catch our children and rape them. You find our girl children pregnant and even do not know who the person responsible for this is. At times you find this pregnancy is even for the father… sincerely speaking men are on* the *rampage. They touch the breasts of girls, squeeze girls tightly without their consent, romancing them forcefully.” P28: IDI_KYA_07*

*“We adults who are working, if you are like a teacher and your fellow teacher who is above you, for example, a* head-teacher*, whenever you go to his office, you think that he has called you for official duties. He may start touching you inappropriately. Even if he does not engage you in sexual intercourse, you see that is his intention. Or if sometimes you still want to keep your job, you accept without being okay with it. You say that let me do what he wants so that I keep my job. But because you still want to survive, if they chase you from your job, getting another one will be difficult, so you decide to do what he wants you to do, and it stresses you.” P31: IDI_KYA_10*

In contrast, others viewed sexual violence as the way men keep looking at females inappropriately. Females being bothered with unsolicited gifts, love letters, phone calls, and love messages were perceived as a form of sexual violence. Men demanding sex for having given basic things of life to women mentioned as being sexual violence.

*“Some men write love letters to women* to woo *them just because you are there and yet you are not interested. Secondly, when he meets you, he wants to always sweet talk, so that you end up liking him and yet you do not like him. Then with time, he will want to bring you a nice gift to drift you from your decision and influence you into doing what he wants.” P15: IDI_KIG_31*

*“He keeps on looking at you for long, he keeps on coercing you as he says so many things, and he mixes in some other good words. Sometimes he makes several phone calls, disorganizes you… sometimes he sends you bad messages with an intention of forcing you into sexual activity.” P 1: IDI_KIG_19*

*“There are times when someone you do not even know starts calling you on phone, he keeps calling and sending you messages that you do not want, but that person insists and shows you that he knows you. Or you find a man is touching your breasts or your butt, so such things happen even this side, someone keeps looking at you as if you owe them something and yet you owe him nothing.” P22: IDI_KYA_01*

#### Effects of sexual violence mentioned by participants

Participants had different views about the effects of sexual violence. These views ranged from physical, biological, social, and mental effects. Respondents pointed out that survivors of sexual violence might become infected with sexually transmitted diseases such as HIV/AIDS. The survivors may conceive unplanned pregnancies with their untoward complications. They pointed out that in cases where force is used, survivors sustain physical trauma like damage to the uterus and private parts. Participants mentioned stigmatization, stress, and depression amongst the survivors of sexual violence that can lead to suicide. Participants pointed out marriage break-up, isolation, failure to associate with others, and low self-esteem as other effects of sexual violence.

*“You find a man infecting his children with HIV, impregnating her, the dangers are so many, now if this girl gets impregnated by his father, which man is going to marry her, yet she is even HIV positive. So, you find that girl getting stressed, lacking peace and getting stigmatized because her father made her pregnant, so she will not get someone to marry her”, P24: IDI_KYA_03*

*“Now*, *for example*, *a person who has been raped … some of them get so much disturbed to the extent that they refuse completely to get married because they completely hate anything that is called a man in all her life just because of being raped.” P27: IDI_KYA_06*

*“She might get pregnant, contract diseases, sometimes they might hurt her and might end up losing her uterus. Sometimes when she gets pregnant, she might want to abort and might end up dying in the process of aborting, or you find her loitering a long road without any form of care, you find her crying, in the end, she commits suicide.” P25: IDI_KYA_04*

#### Awareness about the existence of sexual violence in the community

When the community is not aware that sexual violence occurs, they will not advocate for the services intended to address the problem. Participants stated that sexual violence was in their communities, and it was a big concern. Some of the participants had even witnessed the people suffering from the effects of sexual violence.

*“Sexual violence happens especially in my home areas (name of village mentioned), there is a man that chased his wife away from home, after that, he started to violate his children sexually, and by the way*, *he was arrested by the police.” P24: IDI_KYA_03*

*“You find a man having sex with his daughters sometimes they go ahead to encourage them to use family planning so that they do not get pregnant and give birth to babies so that he can continue having sex with them. These things are happening in our community. We see them.” P10: IDI_KIG_28*

*“You find a lot of young girls in our communities getting pregnant when they were not willing and ready for their pregnancies. If you are to investigate, you find that they were raped.” P33: IDI_KYA_12*

*“That child, her colleagues kept on harassing her saying that, “look at this small child who was raped” and she finally developed self-hatred when they kept on saying that “that small child was raped” she ended up drinking poison and committed suicide.” P 4: IDI_KIG_22*

Some of them shared their lived experiences with sexual violence. Although this was not the primary focus of the study, some of the responses ended being personal.

*“Sexual violence occurs because there is a badly behaved man who usually sends me messages when I am not even willing. We even have a child that I know, a badly behaved man met her there at the well and raped her. ” P 4: IDI_KIG_22*

*“It occurs because personally, a certain man forcefully raped me and made me pregnant.” P 6: IDI_KIG_24*

Contrary views were that sexual violence did not occur in the community, and or if it occurred, it was not common. A few people held this view.

*“Others do not know anything about sexual violence.” P33: IDI_KYA_12*

*“No, in my village it is not so common.” P25: IDI_KYA_04*

### Knowledge about trauma-informed services for survivors of sexual violence

This section of the manuscript describes participants’ knowledge about whether survivors of sexual violence knew where to seek help and if they did seek help. It also intends to find out if the participants knew about different trauma-informed services available. This was done bearing in mind that trauma-informed services are services provided with prior knowledge and experience of the violence so as not to re-traumatize the survivor, and know that health providers had been trained in offering these services. This is important since if services are available but the community is not aware of their presence and/ or does not use them, then they become white elephants.

#### Knowing where to seek trauma-informed services

Participants said that survivors of sexual violence sought help. In most cases, this help was sought from both health facilities and police for treatment and justice respectively. There were mixed views on whether the survivors of sexual violence go first to a health facility or a police station. Those who advocated for the survivors of sexual violence going to the health facility first reasoned that the survivor needed medical attention to address injuries sustained or to prevent infections. On the other hand, proponents of survivors going to the police first were more concerned that delay to report may aid the perpetrator to run away from the place. They mentioned that thorough investigations are made to identify real and fictitious cases of sexual violence. The prosecution is then done and justice is provided.

*“A person who has been sexually assaulted should go and report to the police immediately and then they serve justice. After coming from the police, and if you find you have some health issues then you go to the hospital and you get the treatment that you deserve, and then after you can go back to the police to go ahead with your case so that it can be investigated”. P 8: IDI_KIG_26*

*“They are supposed to go to the hospital both in the private and government hospital, and if it is a child and the case is defilement, the perpetrator will be taken to the people who are fighting for children’s rights, if it is where they chose to go first, they will do what they can and then they forward them ahead for further services.” P10: IDI_KIG_28*

*“They should run to the hospital to carry out a checkup, to see what harm has been caused, in case she has not yet contracted any diseases, they give her medicine to block and stop all possible diseases that she could contract. Some run to the police and report what has happened, so they carry out investigations at the police.” P35: IDI_KYA_14*

In contrast, some participants revealed that some survivors opted not to seek any services in case they are victimized due to the fear of the perpetrator, bribing of the family of the affected and authorities, or even fear of family breakup and stigmatization. To some survivors of sexual violence, it was unheard of that the husband can sexually violate his wife. Some participants reported that they feared being identified as rape survivors as this was very shameful and embarrassing so they would rather not report it anywhere.

*“….you have nothing to do because the whole world knows that he is your husband, you decide to keep quiet. And again you get shy to talk about it, you get fear for disclosure… you let him do what he wants…” P 3: IDI_KIG_21*

Also, participants stated that other survivors of sexual violence did not have someone to share with their experience and ended up not seeking help. Others did not even know where to seek help. On the other hand, some of the survivors of sexual violence lived in fear of death since they were warned that they would be killed should they disclose that they were sexually violated.

*“When it happens to some, they don’t get who to share with or others fear to talk, they decide to keep quiet and keep it at “heart”. Others don’t know who to talk to or where to go, she doesn’t know what she should have done. Some keep silent and they look at it as a shameful act… they think that if they say it they are going to be embarrassed. And they think that if they tell some, they are also going to tell others… and they end up ashamed and bothered.” P 1: IDI_KIG_19*

*Some are being warned by their father saying “the moment you say anything about it, I will kill you”. He instead makes her keep silent and quiet by giving her some gifts. In most cases, you find such children are not fitting properly in their homes just because of that. P27: IDI_KYA_06*

#### Knowledge about available trauma-informed services

Participants were asked to mention different trauma-informed services. Participants pointed out that survivors should report immediately to the health facilities so that they can be examined to see if they have contracted illnesses or injuries and then report to the police to apprehend the perpetrator. A section of the participants mentioned that the services to be received included testing for HIV and other infections. Receiving the prophylaxis treatment for HIV and other infections was also cited. Other participants talked about pills that are given to prevent pregnancy. Participants talked about counseling that is done to comfort the survivors. Respondents stated that those with physical injuries are also provided with the services. Participants cited the legal services like having medico-legal forms completed and assistance obtained from police as one the services. Most of the participants mentioned only the services related to HIV leaving out other services.

*“A person who has been sexually assaulted should go and report to the police immediately and then they serve justice. After coming from the police, and if you find you have some health issues then you go to the hospital and you get the treatment that you deserve and then after you can go back to the police to go ahead with your case so that it can be investigated”, P 8: IDI_KIG_26*

*“At the hospital, the victim is checked to see the extent to which they have been affected, tested to see if they have been infected with HIV, checked to see if they are pregnant, and given treatment to curb any diseases that have been transmitted to the victim. They also provide* counseling *services to the victim to reduce the trauma the crime has caused.” P 7: IDI_KIG_25*

### Attitudes towards trauma-informed services for victims of sexual violence

This section of the manuscript describes the perceptions of participants about the quality of trauma-informed services. Quality is viewed as the extent to which a service satisfies a patient. Quality appreciation is key to the willingness to utilize services by clients. The participants described the quality of trauma-informed services. Quality service is comprehensive, accessible, relevant, efficient, and equitable.

#### Comprehensiveness of available trauma-informed services

A section of participants mentioned that the services are of good quality. Participants said that the services are available. The survivors are examined and given appropriate medication like ARVs, counseling. The services offered gave confidence and hope to survivors of sexual violence. However, most participants did not know about testing and treatment for other sexually transmitted infections and counseling. The majority focused mainly on HIV. The police investigate cases of sexual violence leading to the prosecution of perpetrators. In this way, survivors of sexual violence get justice. They stated that if one goes to any of the facilities, one will get help. Health workers sometimes interrogated survivors and blamed them for being victimized. Some participants mentioned that these services are not all available at the health facilities and survivors get some but not all of them.

*“But generally, the services are good, in the hospital,* there *are available, because when you get at the hospital, you, first of all, get* counseling *and they tell you exactly what to do and what is going on, they tell you each and everything. They counsel you properly, make you gain your confidence and hope. They also show you what you are supposed to do and you go back home very okay, but when you go to the police, they also help you and generally you find every service that you get is very wonderful and you come back home satisfied.” P19: IDI_KIG_34*

### Accessibility of services and equity of services provided

Participants reported that trauma-informed services were available and accessible. Services were free since they were in public health facilities. Most participants considered geographical accessibility to be good.

*“So, when you reach the hospital, and you look for your doctor immediately you tell him or her what has happened, she or will understand the situation quickly and he knows what to do to you. Then he will first give you the treatment that you deserve according to how you have explained to him. Sometimes it may be a very young child and then he will have to first check to find out whether her uterus is okay, then after they test for HIV and they also find out if she is already infected. From there then he or she makes a medical form for you to take to the authorities like police, and police help you to hunt for the perpetrator”, P19: IDI_KIG_34*

In another perspective, some participants reported that the services were not that perfect due to some factors. Participants mentioned events of bribery to the police survivors and not getting justice. The poor road network delays reporting to the respective facilities by the survivors of sexual violence.

*“It also depends how the police officers treat you because when you get there, they treat you anyhow, after being raped, for example, you find they do not care to get to arrest the perpetrator. They do not care to find him, will ask you* where *you were, you feel you hate the services they are trying to give you. But sometimes, you may* get there a *health worker who is not caring. She or he will look at you and ignore you and keep quiet as if you are enemies. She or he may start interrogating you and asking you questions and why you got involved in such actions, can go ahead and abuse you, and start calling you all sorts of names. In the end, you may end up demotivated and go back home without getting any services. Health workers are not the same, may end up making the situation worse than it was.” P23: IDI_KYA_02*

*“Maybe she can just rush to the hospital even some of the health workers want money before they do anything on the victim.” P11: IDI_KIG_29*

## Discussion

This paper explored the knowledge, attitudes towards trauma-informed services for survivors of sexual violence and factors associated with the utilization of these services by females. There is no consensus on the definition of sexual violence. However, most participants perceived the meaning of sexual violence as forcing someone into acts of sexual activities against the will or when someone has not consented. Different forms of sexual violence were mentioned included rape and defilement, forced sex among married women by husbands, unauthorized touching of body parts like breasts and buttocks, verbal violence, and inappropriate looks. The understanding of sexual violence by our study participants was in agreement with the definition of sexual violence by the World Health Organization. Sexual violence is defined as “any sexual act, attempt to obtain a sexual act, unwanted sexual comments or advances, or acts to traffic, or otherwise directed, against a person’s sexuality using coercion, by any person regardless of their relationship to the victim, in any setting, including but not limited to home and work” by World Health Organization [30].

In this study, most of the participants mostly mentioned contracting HIV as the effect of sexual violence. These could have been due to a high prevalence of HIV in the area at 7.9% compared to Uganda’s national average of 6% [4]. The different effects of sexual violence cited in this study were in line with those found in other studies [17–20]. Participants showed varying knowledge about trauma-informed services for survivors of sexual violence. Most participants mentioned different trauma-informed services like HIV testing and treatment and counseling that were in line with the services mentioned by the Uganda Clinical Guidelines, 2016 [22]. Participants mostly mentioned services related to HIV and left out other necessary services like antibiotic prophylaxis, tetanus toxoid, and this pointed out a gap in the knowledge about these services.

In this study, we found out that some survivors of sexual violence did not seek help. This finding was similar to the results of other studies done in low and middle-income countries [24, 31–33]. Women sexually violated by their husbands would not even try to seek trauma-informed services, because it is unheard of in the communities that a husband can sexually abuse his wife. There was a perception that sexual violence was acceptable. This finding was in agreement with other studies done in Uganda [34, 35]. Survivors of sexual violence did not seek help due to fear, stigmatization from the community, and intimidation by the perpetrator. These findings are similar to those found in Uganda’s demographic and health survey, 2011 [24].

Although some participants mentioned that services offered were of good quality and that all the services were accessible from the respective facilities, gaps in services were noted. The highlighted gaps included bribery, limited availability at the health facilities, survivors being interrogated by health workers while seeking the services, survivors not being given the attention and care, and delayed service delivery may be due to the uniqueness of the communities. In health care, actual practices in response to survivors of sexual violence may vary despite the existence of both international and local guidelines. A systemic review done by Gatuguta and others found that health care workers were uncomfortable examining survivors of sexual violence due to confidentiality and lack of clarity of role [23]. Other studies have shown flaws in delivering trauma-informed services by a shortage of doctors [21, 36].

The utilization of trauma-informed services by sexual survivors of violence was taking place. However, it was negatively affected by stigma at the community level, being judged negatively by the community, poor road network, long-distance from health facilities, unreliable or unavailable services, and healthcare professionals’ attitudes. These findings are similar to other studies [37, 38]. These point out the need for sensitization about trauma-informed services and sexual violence for both the community and victims of sexual violence.

### Strengths and limitations

The study participants were females attending a health facility. They might not have experienced sexual violence and thus may not be having the information regarding trauma-informed services for survivors of sexual violence. We counteracted this by including participants of different age groups who provided us with views about trauma-informed services for sexual violence. Participants were encouraged to share with us what was taking place in the community. Also, some of the participants shared lived experiences of sexual violence. In addition, sexual violence is a sensitive topic in the community. Thus, the respondents may have felt compelled to answer questions in a particular manner whether or not it represented their opinions despite being assured of confidentiality and that data would be anonymized. We limited this by using interviewers who had no established contacts with participants before. Nevertheless, this study provides results that can be used with adaption in similar settings to improve the lives of survivors of sexual violence.

## Conclusions

Generally, the participants showed a knowledge gap about trauma-informed services for survivors of sexual violence. Sexual violence was present in the community. The study highlighted barriers to trauma-informed services. There is a need for thorough sensitization about these services and evaluation of different service providers by authorities to improve service delivery. The knowledge, attitudes, and factors associated with the utilization of trauma-informed services should be considered while formulating interventions for survivors of sexual violence. Further studies are needed to document the lived experiences of survivors of sexual violence.

## Data Availability

The datasets generated and/or analyzed during the current study are not publicly available due to the restrictions stated in our study consent forms. However, de-identified data can be made available on reasonable requests made to the corresponding author.

## Abbreviations

ARVs: Antiretroviral therapy
AIDS: Acquired immune deficiency syndrome
HEPI- HEPI-TUITAH: Health professional education partnership initiative -transforming Ugandan institutions training against HIV/AIDS
HIV: Human immune virus
IDI: In-depth interview
KIG: Kigarama
KYA: Kyangyenyi
MUST: Mbarara University of Science and Technology
